# Insights into high-pressure acclimation: comparative transcriptome analysis of sea cucumber *Apostichopus japonicus* at different hydrostatic pressure exposures

**DOI:** 10.1186/s12864-020-6480-9

**Published:** 2020-01-21

**Authors:** Linying Liang, Jiawei Chen, Yanan Li, Haibin Zhang

**Affiliations:** 1 Institute of Deep-sea Science and Engineering, Chinese Academy of Sciences, Sanya, 572000 China; 2 University of Chinese Academy of Sciences, Beijing, 100049 China

**Keywords:** Hydrostatic pressure, Acclimation, Transcriptome, Differentially expressed gene, Sea cucumber

## Abstract

**Background:**

Global climate change is predicted to force the bathymetric migrations of shallow-water marine invertebrates. Hydrostatic pressure is proposed to be one of the major environmental factors limiting the vertical distribution of extant marine invertebrates. However, the high-pressure acclimation mechanisms are not yet fully understood.

**Results:**

In this study, the shallow-water sea cucumber *Apostichopus japonicus* was incubated at 15 and 25 MPa at 15 °C for 24 h, and subjected to comparative transcriptome analysis. Nine samples were sequenced and assembled into 553,507 unigenes with a N50 length of 1204 bp. Three groups of differentially expressed genes (DEGs) were identified according to their gene expression patterns, including 38 linearly related DEGs whose expression patterns were linearly correlated with hydrostatic pressure, 244 pressure-sensitive DEGs which were up-regulated at both 15 and 25 MPa, and 257 high-pressure-induced DEGs which were up-regulated at 25 MPa but not up-regulated at 15 MPa.

**Conclusions:**

Our results indicated that the genes and biological processes involving high-pressure acclimation are similar to those related to deep-sea adaptation. In addition to representative biological processes involving deep-sea adaptation (such as antioxidation, immune response, genetic information processing, and DNA repair), two biological processes, namely, ubiquitination and endocytosis, which can collaborate with each other and regulate the elimination of misfolded proteins, also responded to high-pressure exposure in our study. The up-regulation of these two processes suggested that high hydrostatic pressure would lead to the increase of misfolded protein synthesis, and this may result in the death of shallow-water sea cucumber under high-pressure exposure.

## Background

The ocean is warming because of global climate change, forcing the bathymetric migrations of shallow-water marine invertebrates [1, 2]. As such, the ability of a shallow-water invertebrate to acclimatize to deep-sea environments during its lifetime is vital. The bathymetric migrations of marine fauna are predicted to be constrained by the combined effects of temperature, hydrostatic pressure, and oxygen concentration [2]. Among them, hydrostatic pressure is thought to be the major environmental factor that limits the vertical distribution of extant marine fauna [3, 4]. Many studies have examined the tolerance of shallow-water invertebrates to high hydrostatic pressure and low temperature (reviewed by Brown & Thatje 2014) [5], indicating that many extant marine benthic invertebrates can tolerate hydrostatic pressure outside their known natural distributions, and a low temperature can impede high-pressure acclimation. Although a few studies focused on DEGs responding to high-pressure exposure [6–8], transcriptome analysis was seldom applied to relevant studies, and the molecular mechanisms of shallow-water invertebrates to acclimatize to high-pressure environment is not yet fully understood. This question is important in the present context of climate change and ocean warming.

Most extant deep-sea fauna are accepted to have originated from shallow waters as a consequence of a series of extinction events during the Phanerozoic [9, 10]. The colonization of the deep sea occurs throughout selection and during the slow genetic drift of species that gradually adapt to life in this area [5], whereas the high-pressure acclimation of shallow-water fauna involve physiological plasticity in response to a simulated immersion in the high-pressure environments. However, both evolutionary adaptation and phenotypic acclimation are essential for adaptation to high pressure [11]. Transcriptome analysis has been applied widely to study the adaptation mechanisms of deep-sea fauna based on the comparisons of congeneric species that have different vertical distribution profiles. Common adaptation patterns have been observed in different taxa of deep-sea living fauna [12]. Many biological processes, including alanine biosynthesis [13], antioxidation [14, 15], energy metabolism [13, 16], immunity [16, 17], fatty acid metabolism [18], and genetic information processing [13], are related to deep-sea adaptation.

Somero (1992) has reviewed the effects of hydrostatic pressure on shallow-water organisms [19]. One of the most sensitive molecular assemblages of hydrostatic pressure is lipid bilayer [11, 19–22]. High pressure leads to a reduction of membrane fluidity, impeding physiological membrane functions, such as transmission [20, 23], transmembrane transportation, and cell movement [24, 25]. The effects of high hydrostatic pressure and low temperature are similar [26, 27]. Parallel effects can be detected on the basis of membrane composition with an increase in hydrostatic pressure of 100 MPa and a reduction in temperature of 13–21 °C [19]. A high hydrostatic pressure causes the depolymerization of protein structures, whereas a low temperature negatively affects protein activity, and both factors induce an increase in protein chaperoning, thereby decreasing the stabilization of secondary RNA and DNA structures [28, 29]. High pressure can also strengthen hydrogen bonds. Consequently, processes that include DNA replication, transcription, and translation are impeded [30, 31].

The sea cucumber *Apostichopus japonicus* (phylum: Echinodermata) is a temperate species mainly distributed along the coastal area of eastern Asia [32]. It is also a popular food in China because of its high nutritional and medicinal value. Sea cucumbers of Echinodermata are not only ubiquitous in coastal areas but also widespread at abyssal depth [33, 34]. Since deep-sea species do not obtain new genes, but utilize gene sets homologous to their coastal relatives to adapt to deep-sea environments [18], we predicted that *A. japonicus* has the potential to acclimatize to high-pressure environment, and used this species in high-pressure incubations. A pressure vessel was used to perform high-pressure exposure on experimental samples, provide a stable and controllable experimental context, and examine pressure acclimation accurately [35].

## Results

### Hydrostatic pressure tolerance of *A. japonicus* and experimental design

To examine the pressure tolerance of *A. japonicus*, we incubated 10 individuals at different high-pressure conditions and measured their mortality rate before formal experiments for transcriptome analysis. There were 30% individuals died after 24-h incubation at 35 MPa, but no individual died at 25 MPa. Additionally, eversion was not observed at 25 MPa, which is usually happened when sea cucumbers are stressed. Consequently, 3 pressure conditions were set: 0.1 MPa (atmospheric pressure), 15 MPa (pressure at the depth of 1500 m), and 25 MPa (pressure at the depth of 2500 m). A total of 9 individuals (3 individuals from each experimental group) were high-pressure incubated for transcriptome analysis. The RNA of body wall tissue from each individual was sequenced, and paired reads of these 9 samples were assembled into one tanscriptome.

### Sequencing, assembly and annotation

Three experimental groups (P0.1, experimental group incubated at 0.1 MPa; P15, experimental group incubated at 15 MPa; and P25, experimental group incubated at 25 MPa) were used for comparative transcriptome analysis. Each experimental group had three replications. Sequencing qualities are listed in Additional file 2: Table S1. Paired reads from the nine samples were assembled into 553,507 unigenes with a total length of 481,946,001 bp and an N50 length of 1204 bp. BUSCO completeness of the transcriptome were 91.5% (single-copy: 28.4%, duplicated: 63.1%, fragmented: 7.2%, missing: 1.3%). There were 14, 23, and 7% unigenes annotated in the databases of Swiss-Prot, Protein family (Pfam), and Kyoto Encyclopedia of Genes and Genomes (KEGG), respectively.

### DEGs involved in high-pressure acclimation

**Fig. 1:**
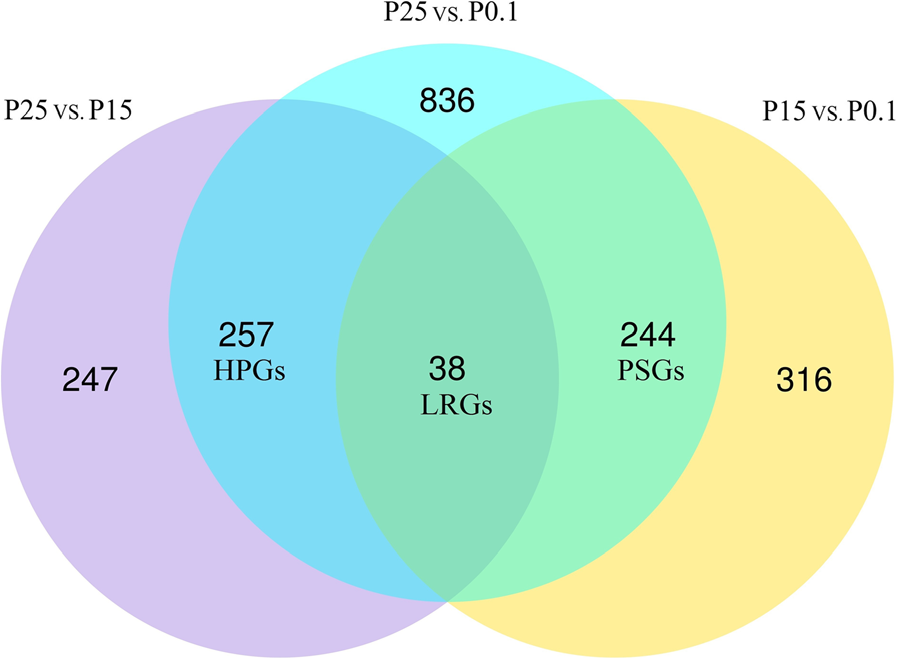
Venn diagram of DEGs among different combinations (P15 vs. P0.1, P25 vs. P0.1, and P25 vs. P15). P0.1: experimental group incubated at atmospheric pressure; P15: experimental group incubated at 15 MPa; P25: experimental group incubated at 25 MPa; DEGs: differentially expressed genes; LRGs: linearly related DEGs; PSGs: pressure-sensitive DEGs; HPGs: high-pressure-induced DEGs

Three combinations, namely, P15 vs. P0.1, P25 vs. P0.1, and P25 vs. P15, were subjected to differential expression analysis by using the DESeq2 R package (v1.22.2) [36]. In this study, up-regulated genes were considered as activated genes in response to high-pressure exposure because only essential processes can be maintained, whereas nonessential processes are reduced outside the optimal range [37–40]. A total of 598 genes, 1375 genes, and 542 genes were significantly up-regulated in the combinations of P15 vs. P0.1, P25 vs. P0.1, and P25 vs. P15, respectively (Fig. 1). In addition, quantitative real-time reverse transcription-PCR (qPCR) analysis was used to validate the reliability of the RNA-seq results. A total of 14 DEGs were employed for qPCR analysis, and the Pearson correlation coefficients between RNA-seq and qPCR results ranged from 0.81 to 0.99.

**Fig. 2:**
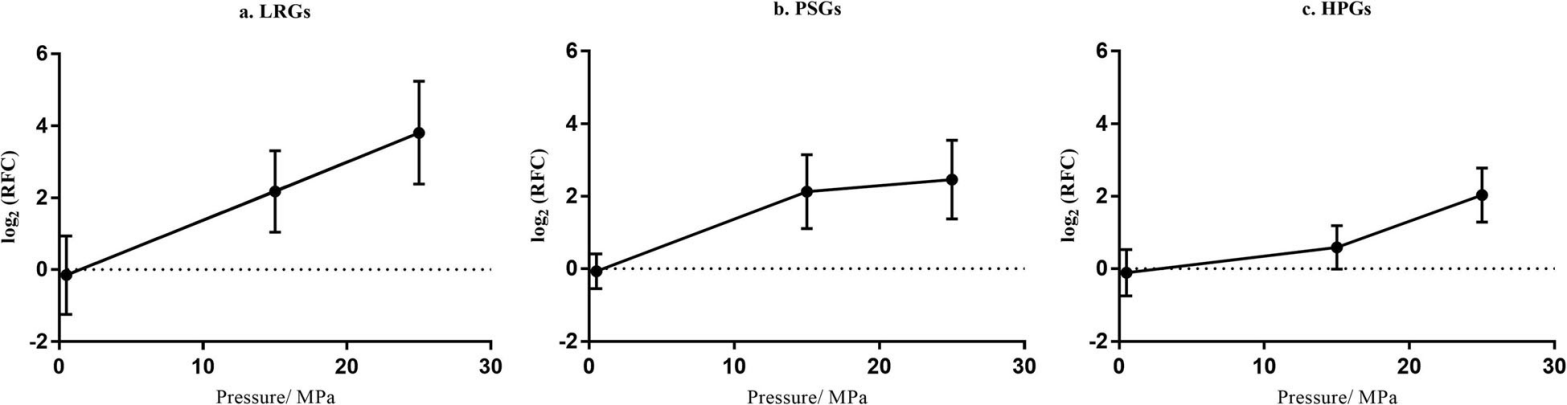
Line graphs of the expression patterns of LRGs, PSGs, and HPGs. Points represent the mean of log_2_ (RFC) of all genes. Error bars represent standard deviation. LRGs: linearly related DEGs; PSGs: pressure-sensitive DEGs; HPGs: high-pressure-induced DEGs; RFC: relative fold change

Three groups of DEGs comprising 38 linearly related DEGs (LRGs), 244 pressure-sensitive DEGs (PSGs), and 257 high-pressure-induced DEGs (HPGs) (Fig. 1) were identified according to their gene expression patterns. LRGs were up-regulated among the three combinations. PSGs were up-regulated only in P15 vs. P01 and P25 vs. P01. HPGs were up-regulated only in P25 vs. P01 and P25 vs. P15. The expression pattern of LRGs was linearly correlated with hydrostatic pressure (R^2^ > 0.99, Fig. 2a). The PSGs were significantly up-regulated at 15 MPa and remained at a similar high level at 25 MPa (Fig. 2b). The HPGs were significantly up-regulated at 25 MPa but were not significantly up-regulated at 15 MPa (Fig. 2c).

### Swiss-Prot annotation of LRGs, PSGs, and HPGs

**Fig. 3:**
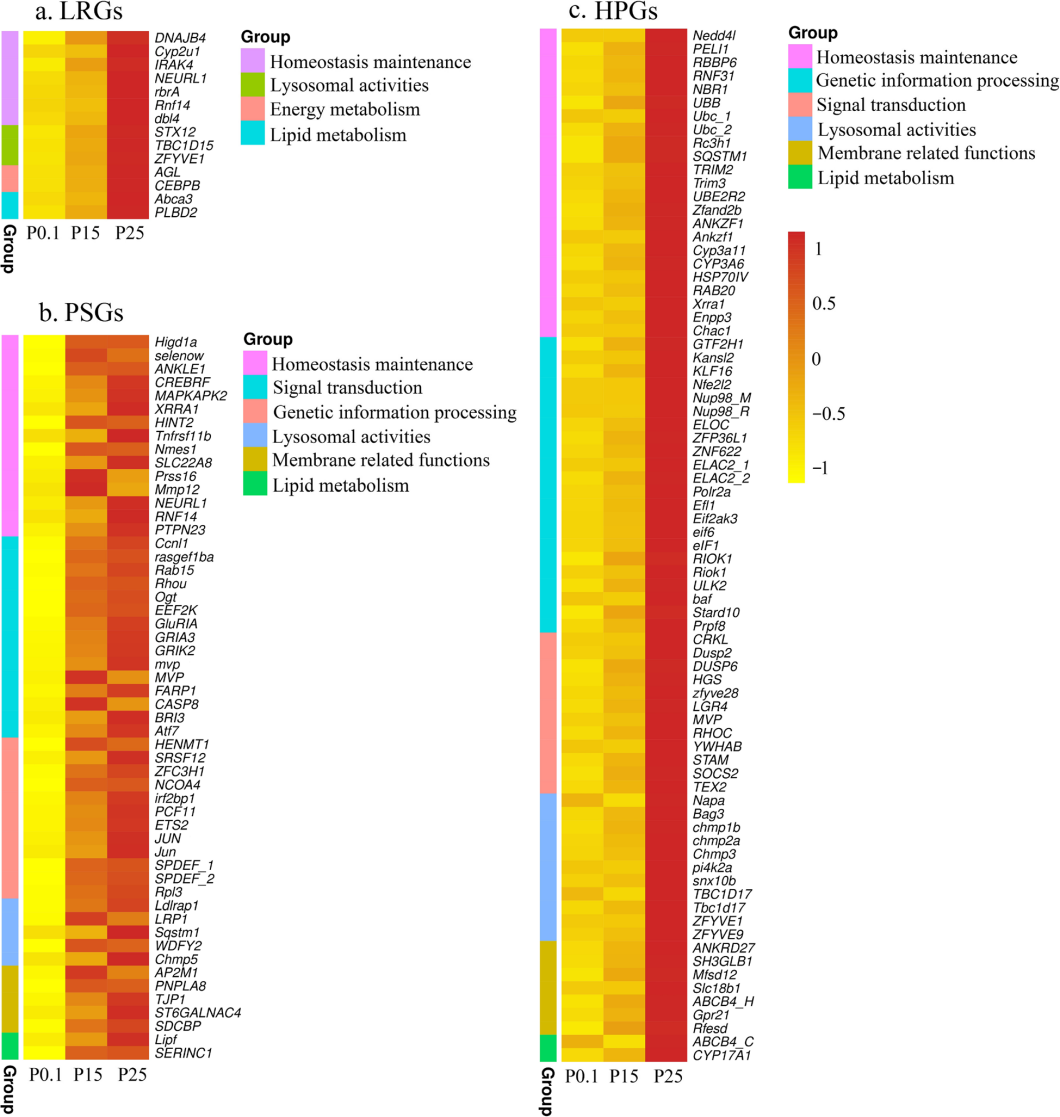
Heatmaps of DEGs annotated in Swiss-Prot. **a** Heatmap of linearly related DEGs. **b** Heatmap of pressure-sensitive DEGs. **c** Heatmap of high-pressure-induced DEGs. P0.1: experimental group incubated at atmospheric pressure; P15: experimental group incubated at 15 MPa; P25: experimental group incubated at 25 MPa; DEGs: differentially expressed genes

The expression patterns of 38 DEGs are linearly related to hydrostatic pressure, and 14 of them are annotated in the Swiss-Prot database (Additional file 3: Table S2). Their functions are mainly involved in homeostasis maintenance (7 genes) and lysosomal activities (3 genes) (Fig. 3a). Four of the seven homeostasis maintenance genes, namely, E3 ubiquitin-protein ligase NEURL1 (*NEURL1*), E3 ubiquitin-protein ligase RNF14 (*RNF14*), E3 ubiquitin-protein ligase dbl4 (*dbl4*), and E3 ubiquitin-protein ligase rbrA (*rbrA*), are involved in ubiquitination. The three other genes involved in homeostasis maintenance are DnaJ homolog subfamily B member 4 (*DNAJB4*), cytochrome P450 2 U1 (*Cyp2u1*), and interleukin-1 receptor-associated kinase 4 (*IRAK4*). DnaJ, also known as heat shock protein 40, is a molecular chaperone protein regulating the ATPase activity of heat shock protein 70 (HSP70) [41]. Cytochrome P450 proteins (CYPs) are known for their antioxidative functions [42]. The IRAK4 protein is a key regulatory kinase of innate immunity [43]. Three genes, namely, syntaxin-12 (*STX12*) that regulates protein transport between late endosomes and the *trans*-Golgi network, TBC1 domain family member 15 (*TBC1D15*) that promotes fusion events between late endosomes and lysosomes [44], and zinc finger FYVE domain-containing protein 1 (*ZFYVE1*) that has been related to vacuolar protein sorting and endosome function, are implicated in lysosomal activities. Two genes, namely, CCAAT/enhancer-binding protein beta (*CEBPB*) that regulates the glucose homeostasis [45] and glycogen debranching enzyme (*AGL*) that facilitates the breakdown of glycogen and serves as glucose storage, participate in energy metabolism [46]. Two genes, namely, ATP-binding cassette sub-family A member 3 (*Abca3*) [47] and putative phospholipase B-like 2 (*PLBD2*), function in lipid metabolism.

A total of 244 genes are PSGs, and 70 of them were annotated in Swiss-Prot database (Additional file 4: Table S3). These 70 genes were grouped into seven different biological processes, namely, homeostasis maintenance (15 genes), signal transduction (15 genes), genetic information processing (12 genes), lysosomal activities (5 genes), membrane related functions (5 genes), lipid metabolism (2 genes), and others (16 genes) (Fig. 3b). Of the 15 genes grouped in homeostasis maintenance, 6 are involved in stress responses, including ankyrin repeat and LEM domain-containing protein 1 (*ANKLE1*) involving DNA damage response and DNA repair, CREB3 regulatory factor (*CREBRF*) involving unfolded protein response, and MAP kinase-activated protein kinase 2 (*MAPKAPK2*) involving cell migration, cell cycle control, DNA damage response, and transcriptional regulation; 6 are implicated in immune response, including histidine triad nucleotide-binding protein 2 (*HINT2*) involving apoptosis; and 3 participate in ubiquitination. Of the 12 genes grouped in genetic information processing, 7 function in transcription.

A total of 257 genes are HPGs, and 123 of them were annotated in Swiss-Prot database (Additional file 5: Table S4). These genes were grouped into six different biological processes, namely, homeostasis maintenance (23 genes), genetic information processing (22 genes), signal transduction (12 genes), lysosomal activities (11 genes), membrane related functions (7 genes), lipid metabolism (2 genes) and others (46 genes) (Fig. 3c). Of the 23 genes grouped in homeostasis maintenance, 13 are involved in ubiquitination, including ubiquitin-conjugating enzyme E2 R2 (*UBE2R2*), E3 ubiquitin-protein ligase *NEDD4*, *PELI1*, *RBBP6*, and *RNF31*; 8 are implicated in stress response, including cytochrome P450 *Cyp3a11* and *CYP3A6*, heat shock 70 protein IV (*HSP70IV*), AN1-type zinc finger protein 2B (*Zfand2b*), ankyrin repeat and zinc finger domain-containing protein *ANKZF1* and *Ankzf1*; and 2 participate in immune response. *Zfand2b* is a recently identified heat shock protein [48]. *ANKZF1* and *Ankzf1* play a role in the cellular response to hydrogen peroxide. Of the 22 genes grouped in genetic information processing, 12 and 7 are involved in transcription and translation, respectively.

### KEGG and Pfam enrichment analysis

**Fig. 4:**
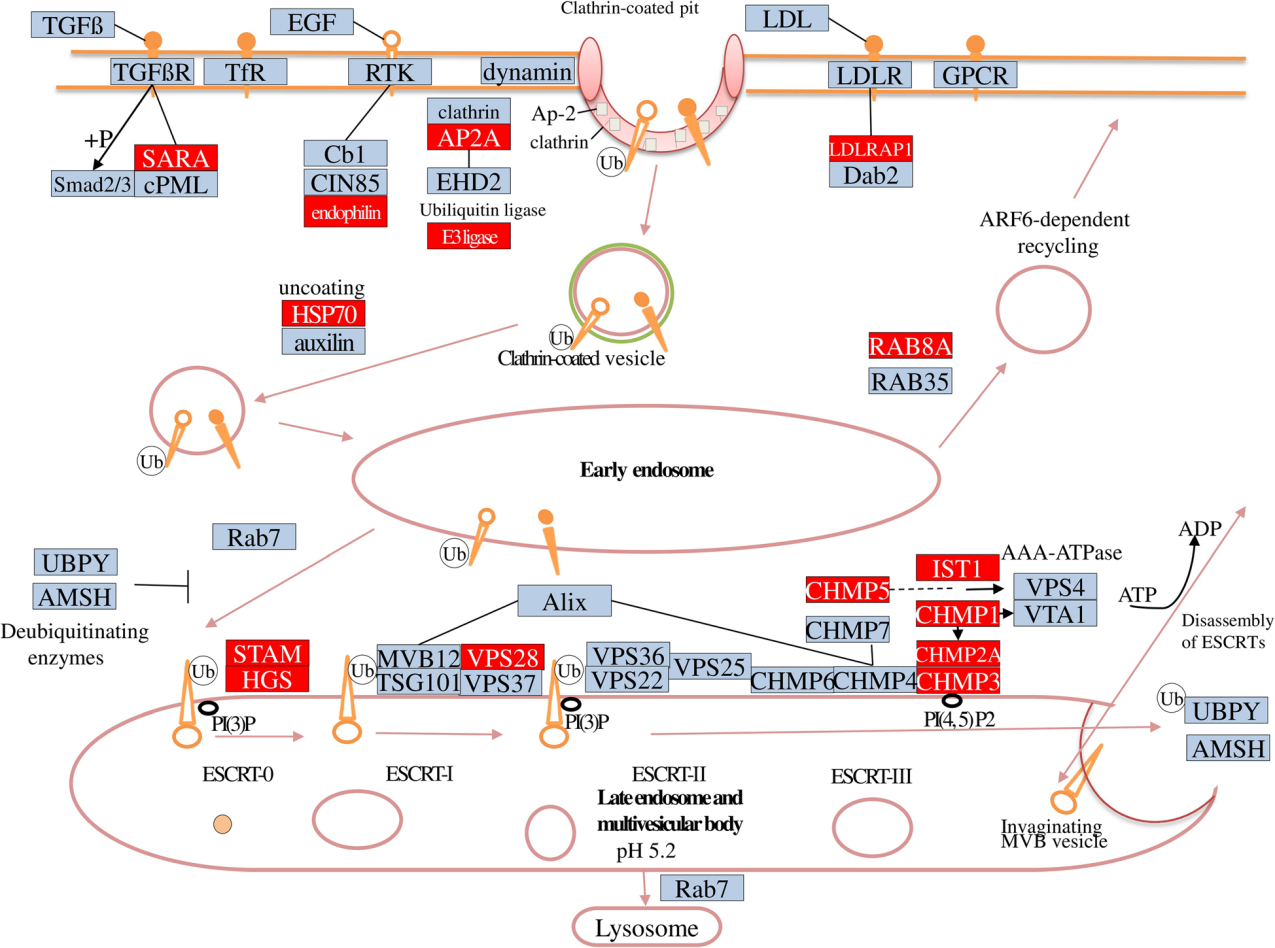
Pathway of clathrin-dependent endocytosis. This pathway is a part of KEGG pathway map (map04144). The proteins involved in this pathway are shown in boxes and their descriptions are listed in the Additional file 6: Table S5. The proteins significantly up-regulated at high-pressure condition in our results are highlighted in red boxes

The KEGG enrichment analysis of LRGs, PSGs, and HPGs were separately implemented by using the KOBAS software [49]. No significantly enriched KEGG pathway existed in any groups of genes except the pathway of endocytosis in HPGs. A total of 14 genes were annotated in this KEGG pathway. Additionally, KEGG enrichment analysis was applied to 539 genes of the assemblage of LRGs, PSGs, and HPGs. Endocytosis was also the most significantly enriched KEGG pathway (Additional file 1: Figure S1). A total of 17 genes were annotated in this KEGG pathway, and most of them were involved in clathrin-dependent endocytosis (Fig. 4 and Additional file 6: Table S5).

**Fig. 5:**
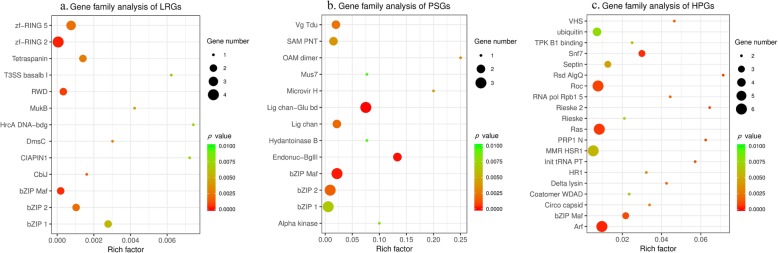
The statistics of gene family analysis. **a** Gene family analysis of linearly related DEGs. **b** Gene family analysis of pressure-sensitive DEGs. **c** Gene family analysis of high-pressure-induced DEGs. DEGs: differentially expressed genes

The Pfam enrichment analysis of LRGs, PSGs, and HPGs were separately implemented by using fisher.test function of R software [50] in LRGs, PSGs, and HPGs. A total of 13, 13 and 20 gene families were significantly enriched in LRGs, PSGs, and HPGs, respectively (Fig. 5).

A total of 13 gene families were significantly enriched in LRGs (Fig. 5a and Additional file 7: Table S6). Three of them, namely, bZIP Maf transcription factor (bZIP Maf), bZIP transcription factor (bZIP 1), and basic region leucine zipper (bZIP 2), are involved in transcription. Two gene families, namely, ring finger domain (zf-RING 2) and zinc-RING finger domain (zf-RING 5), are implicated in the ubiquitination pathway. Two gene families, namely, cytokine-induced anti-apoptosis inhibitor 1apoptosis inhibitor 1 (CIAPIN1) and winged helix-turn-helix transcription repressor (HrcA DNA-bdg), participate in oxidative stress and heat-shock stress response, respectively.

A total of 13 gene families were significantly enriched in PSGs (Fig. 5b and Additional file 8: Table S7). Five of them were involved in transcription (bZIP Maf, bZIP 1, bZIP 2, vestigial family [Vg Tdu], and sterile alpha motif domain [SAM PNT]). Two gene families, namely, ligated ion channel L-glutamate- and glycine-binding site (Lig chan-Glu bd) and ligand-gated ion channel (Lig chan), are implicated in transmembrane ion transportation. The Mus7/MMS22 family (Mus7) participates in DNA damage repair.

A total of 20 gene families were significantly enriched in HPGs (Fig. 5c and Additional file 9: Table S8). Six of them are involved in genetic information related functions. RNA polymerase Rpb1 domain 5 (RNA pol Rpb1 5) catalyzes DNA-dependent RNA polymerization. 50S ribosome-binding GTPase (MMR HSR1) is required for the complete activity of a protein interacting with the 50S ribosome. Rit1 DUSP-like domain (Init tRNA PT) participates in the initiation and elongation of translation. PRP1 splicing factor (PRP1 N) is implicated in mRNA splicing. The regulator of RNA polymerase sigma subunit (Rsd AlgQ) and bZIP Maf function in transcription. Four gene families participate in endocytosis, including ADP ribosylation factor (Arf), Snf7, VHS protein domain (VHS) and coatomer WD associated region (Coatomer WDAD).

## Discussion

The optimum temperature of *A. japonicus* ranges from 10 °C to 17 °C [31], and *A. japonicus* hibernates in winter. The characteristics of *A. japonicus* in hibernation states were quite different from higher animals, but more closely resembled a semi-dormant state. The shift from normal to hibernation was a chronic process, indicated by the gradual depression of metabolic rate of about 71.7% [51]. The water temperature nearly stays constant at 2 °C below the depth of 2000 m [52]. As such, this species is not likely to survive in the deep-sea environments because of the low temperature. However, the scientific question of this study is how shallow-water invertebrates acclimatize to high-pressure environment, and we suggested that the acclimation mechanisms identified in the species *A. japonicus* are similar to other sea cucumber species. Thus we did not simulate the same environments as the deep sea in this study, but examined the molecular responses of *A. japonicus* to high-pressure exposures at 15 °C to prevent variation caused by hibernation, and set hydrostatic pressure as the only variation.

Homeostatic effort is required to maintain internal conditions within their physiological tolerance boundaries outside optimum. Consequently, only essential processes can be maintained, whereas nonessential processes are reduced [37–40]. Survival under such condition is time limited. Although *A. japonicus* can survive at 25 MPa for 24 h, whether it can survive at such pressure condition for longer time is currently unclear. New, et al. (2014) found that the acclimation period of shallow-water shrimp *Palaemonetes varians* to high-pressure condition was 1 week [53]. Thus a long-term high-pressure incubation (1–4 weeks) of *A. japonicus* can provide information to answer this question. However, since the pressure system in used was isolated, we only incubated *A. japonicas* for 24 h to avoid the deterioration of water qualities. The 24-h high-pressure incubation in this study is a first approach. Long-term and time-series high-pressure exposures are the future goal to fully address the molecular mechanisms of *A. japonicus* to acclimatize to high-pressure exposure.

Although LRGs, PSGs, and HPGs have different expression patterns, their up-regulated biological processes are similar. The biological process homeostasis maintenance has the highest proportion in the three groups of DEGs. Additionally, representative biological processes, such as antioxidation, stress response, and immune response, are relevant in many other studies about deep-sea adaptation; similarly, some representative genes, such as HSPs, CYPs, and zinc finger protein, are also involved in deep-sea adaptation [13, 15–18, 54]. It has been proved that the ability of antioxidation can be beneficial to high pressure adaptation: the bacterium *Shewanella piezotolerans* mutant OE100, which enhanced antioxidant defense capacity by experimental evolution under H_2_O_2_ stress, has better tolerance to high pressure [14]. HSPs were also reported to play important role in the maintenance of protein structure which is highly influenced by high pressure [16]. However, DEGs involved in ubiquitination observed in this study were not identified in most relevant studies about deep-sea adaptation. Three enzymes are involved in ubiquitination, including E1 ubiquitin-activating, E2 ubiquitin-conjugating, and E3 ubiquitin-ligating enzymes. Most DEGs participating in ubiquitination in our results were annotated as E3 ubiquitin ligase of RING domin type. E3 ligases can recognize target substrates and facilitate the transfer of ubiquitin from an E2 ubiquitin-conjugating enzyme to its substrate. The number of ubiquitin transferred to substrate can be multiple. Therefore, these modifications can have diverse effects on the substrate, including proteasome-dependent proteolysis, modulation of protein function, structure, assembly, and localization (reviewed by Deshaies & Joazeiro, 2009 [55]).

Endocytosis is the most significantly enriched KEGG pathway in this study. Endocytosis in eukaryotic cells is characterized by the continuous and regulated formation of prolific numbers of membrane vesicles at the plasma membrane [56]. In general, these vesicle types result in the delivery of their contents to lysosomes for degradation. Studies on deep-sea mussels have reported that endocytosis is essential for the acquisition of symbionts [16, 18]. As such, this process has been expanded to the mussel genome. Therefore, we assumed that high pressure could accelerate the development of a deep-sea symbiotic system. Additionally, one of the effects of protein ubiquitination is proteasome-dependent proteolysis, which can activate the following endocytosis. Ubiquitination and endocytosis can collaborate with each other and regulate the elimination of misfolded proteins which resulted from high hydrostatic pressure. The significant up-regulation of these two processes suggested that high hydrostatic pressure would lead to the increase of misfolded protein synthesis, and this may be one of the main reasons resulting in the death of shallow-water sea cucumber under high-pressure exposure.

Gene families involving genetic information related functions, especially transcription, were highly enriched in the three groups of DEGs. Since high pressure can strengthen hydrogen bonds and impedes genetic information related processes [30, 31], the up-regulation of these genes can remit the effects of high pressure. Additionally, genes related to this process were also significantly positive selected in deep-sea amphipod *Hirondellea gigas* [13]. This study suggested that low temperature in deep-sea environments results in the positive selection of these gene families. However, the incubation temperature in our experiments was optimal. We assumed that high pressure also plays an important role in the positive selection of gene families related to genetic information processing. High pressure can cause DNA chain breakage and damage [57]. Thus, high frequencies of DNA repair are needed. The gene family Mus7 and the genes *ANKLE1* and *MAPKAPK2* that participate in the repair of replication-associated DNA damage were also found significantly up-regulated at high-pressure condition in our study.

## Conclusions

Shallow-water sea cucumber *A. japonicus* could survive 100% under 25 MPa at 15 °C for at least 24 h. However, whether this shallow-water species could survive at this high-pressure condition for more than 24 h or permanently remained unclear. The 24-h high-pressure incubation in this study is a first approach. Long-term and time-series high-pressure exposures are the future goal to fully address high-pressure acclimation mechanisms.

Although LRGs, PSGs, and HPGs had different expression patterns, their up-regulated biological processes are similar. Our results also indicated that genes and biological processes involving high-pressure acclimation were similar to those related to deep-sea adaptation. Representative biological processes, including antioxidation, stress response, genetic information processing, and DNA repair, were found significantly induced by high-pressure exposure in our results. In addition, some representative genes, such as HSPs, CYPs, and zinc finger protein, were also detected at high pressure. Moreover, two important biological processes, namely, ubiquitination and endocytosis, which can collaborate with each other and regulate the elimination of misfolded proteins, were found significantly induced by high-pressure exposure. The significant up-regulation of these two processes suggested that high hydrostatic pressure would lead to the increase of misfolded protein synthesis, and this may result in the death of shallow-water sea cucumber under high-pressure exposure.

## Methods

### Collection, maintenance, and high-pressure incubation of *A. japonicus*

Juvenile individuals of *A. japonicus* (length: 5 ± 1 cm, weight: 3.9 ± 0.5 g) were caught from an aquaculture farm in Shandong, China on December 2017. The length was measured in sea water when individuals were under normal activities, and wipe-dry living individuals were used for weight measurement. They were maintained at a closed recirculating aquacultural system (seawater was partially refreshed twice a week). The sea cucumber were reared in aerated filtered seawater (salinity: 34.7–35.3, 1 μm filtered, natural light cycle), and were fed with algae powder twice a week; unconsumed food was removed after 24 h via refreshing seawater. Experimental individuals were starved for 3 d prior to pressurization. The experimental individuals were maintained in the aquacultural system at 15 °C for 2 weeks to acclimatize them to laboratory environments and allow them to recover from collection and handling stress.

The hydrostatic pressure system was set to 15 °C by using circulating water bath at least 6 h prior to each experiment. Three individuals were placed inside the stainless pressure chamber (volume: ~ 20 l, internal diameter 20 cm, internal depth 65 cm) [35] which is full of filtered seawater at a constant temperature (15 ± 0.8 °C), and maintained for 1 h to allow acclimation and recovery from handling stress. Then, the pressure vessel was pressurized at 1 MPa per minute to experimental pressure by using hydraulic pump. The pressure chamber was sealed and isolated during this time period. After hydrostatic pressure exposure for 24 h, the pressure was released instantaneously, and these 3 high-pressure incubated individuals were removed from the pressure chamber and snap frozen in liquid nitrogen. The maximum time elapsed between departure from experimental pressure and flash freezing is 3 min. The flash-frozen individuals were then stored at − 80 °C for further RNA extraction.

YSI Professional Plus (YSI Inc., USA) and HACH DR 1900 (HACH Company, USA) were used to ascertain the stabilization of seawater quality before and after each experiment. No significant difference was found between the experimental context before pressurization (dissolved oxygen: 5.76 ± 0.3 mg.L^− 1^, salinity: 35.0 ± 0.3, pH: 8.1 ± 0.1, NO_2_-N: 0.0055 ± 0.0005 mg.L^− 1^, NH_3_-N: 0.015 ± 0.005 mg.L^− 1^, NO_3_-N: 0.015 ± 0.005 mg.L^− 1^) and after pressurization (dissolved oxygen: 5.50 ± 0.4 mg.L^− 1^, salinity: 35.0 ± 0.3, pH: 8.1 ± 0.1, NO_2_-N: 0.007 ± 0.0005 mg.L^− 1^, NH_3_-N: 0.03 ± 0.005 mg.L^− 1^, NO_3_-N: 0.015 ± 0.005 mg.L^− 1^).

### RNA extraction, sequencing, assembly and annotation

Details of this part have been described in our previous study [35]. In brief, approximately 50 mg body wall tissue from each individual was used for RNA extraction because body wall is easy to be separated from the frozen main body and this tissue is also commonly used for sea cucumber RNA extraction [58]. The tissue was dissected before melted, and immediately transferred into 1 ml of QIAzol (from RNeasy Plus Universal Kit) and total RNA was extracted by RNeasy Plus Universal Kit (QIAGEN, UK) according to the manufacturer’s protocol. A total of 1.5 μg RNA per sample was used for the RNA sample preparations. Then, library preparations were sequenced on Illumina Hiseq X platform and 150 bp paired-end reads were generated. Transcriptome assembly was accomplished based on the clean data by using Trinity [59]. At last, transcripts were hierarchical clustering by Corset [60]. BUSCO (v.3.0.2) was used to estimate the completeness of the final assembly with default settings and using the metazoan orthologs. All unigenes were annotated in three databases, namely, Swiss-Prot, Pfam and KEGG, by using diamond (v0.8.22), HMMER (v3.0) and KEGG Automatic Annotation Server (KAAS), respectively.

### Differential expression analysis

FPKM and read count were calculated by using RSEM [61], and FPKM were normalized by using TMM method. Then, differential expression analysis was implemented by using DESeq2 R package (v1.22.2) [36] to identify DEGs involved in high-pressure acclimation. Only genes with an adjusted *p*-value < 0.01 and |log2 (fold change)| > 1 were regarded as DEGs. In this study, up-regulated genes were considered as activated genes in response to high-pressure exposure because only essential processes can be maintained, whereas nonessential processes are reduced outside the optimal range [37–40]. The relationships of DEGs among different combinations (P15 vs. P0.1, P25 vs. P0.1, and P25 vs. P15) were visualized using the VennDiagram R package (v1.6.20) [62]. The normalized FPKM of each gene were scaled to the control treatment (P0.1) and converted to the relative fold change (RFC). Then, log_2_ (RFC) was used to evaluate differential expression patterns. The expression patterns of LRGs, PSGs, and HPGs were shown on line graphs by using Graphpad Prism 7 (Graphpad software, Inc., USA). Points represented the mean of log_2_ (RFC) of all genes, and error bars represent standard deviation.

The LRGs, PSGs, and HPGs annotated in Swiss-Prot were grouped in several biological processes. Their normalized FPKM, except DEGs grouped into “others”, were visualized by using the pheatmap R package (v1.0.12) [63]. The distance between rows was calculated with vegan R package (v2.5–5) [64] by using the bray method. Pfam and KEGG enrichment analyses (based on Fisher’s exact test) were implemented to identify enriched biological processes in each experimental group by using fisher.test function of R software and KOBAS software [49], respectively. The results of enrichment analysis were visualized by using the ggplot2 R package (v3.2.1) [65].

### RNA-seq validation by qPCR

A total of 14 DEGs were employed for qPCR by StepOnePlus Real-Time PCR System (Applied Biosystems, USA) to validate the RNA-seq results. The primer sequences (Additional file 10: Table S9) were designed by Primer Premier 5.0 software (Premier Biosoft International, Palo Alto, CA, USA). Details of this part have been described in our previous study [35]. In brief, relative standard curve method was used for expression level analysis with *cytb* and *β-actin* as internal controls. Six 3-fold serial dilutions were performed on a cDNA template. The melting curve analysis was performed at the end of each PCR to confirm only one PCR product was amplified. At last, Pearson correlation coefficients between RNA-seq and qPCR results were calculated. Pearson correlation coefficients (ranging from 0.81 to 0.99) are supplied in Additional file 10: Table S9, which validated the reliability of the RNA-seq results.

## Supplementary information

**Additional file 1 Figure S1.** Results of KEGG enrichment.

**Additional file 2 Table S1.** Quality of sequencing. P0.1: experimental group incubated at atmospheric pressure; P15: experimental group incubated at 15 MPa; P25: experimental group incubated at 25 MPa.

**Additional file 3 Table S2.** Swiss-Prot annotation of linearly related DEGs. DEGs: differentially expressed genes.

**Additional file 4 Table S3.** Swiss-Prot annotation of pressure-sensitive DEGs. DEGs: differentially expressed genes.

**Additional file 5 Table S4.** Swiss-Prot annotation of high-pressure-induced DEGs. DEGs: differentially expressed genes.

**Additional file 6 Table S5.** DEGs involved in the pathway clathrin-dependent endocytosis. DEGs: differentially expressed genes.

**Additional file 7 Table S6.** Gene family analysis results of linearly related DEGs. DEGs: differentially expressed genes.

**Additional file 8 Table S7.** Gene family analysis results of pressure-sensitive DEGs. DEGs: differentially expressed genes.

**Additional file 9 Table S8.** Gene family analysis results of high-pressure-induced DEGs. DEGs: differentially expressed genes.

**Additional file 10: Table S9.** Information of the primers used in quantitative real-time reverse transcription-PCR (qPCR) analysis and the PCC between RNA-seq and qPCR results (with cytb and ß-actin as internal control). PCC: Pearson correlation coefficients.

## Data Availability

Relevant data supporting this manuscript are contained within the figures of this manuscript or provided in the supplementary material. The clean data of transcriptome were available from National Center for Biotechnology Information Sequence Read Archive database (SRA accession numbers: SRR8269388, SRR8269389, SRR8269390, SRR8269391, SRR8269384, SRR8269385, SRR8269386, SRR8269387, SRR8269383). The transcriptome assembly project has been deposited at DDBJ/ENA/GenBank under the accession GHDI00000000. The version described in this paper is the first version, GHDI01000000. Some R scripts used in this study have been uploaded to GitHub: https://github.com/CHEN-Jiawei-Jason/R_scripts_for_BMC_Genomics.git

## Abbreviations

DEGs: Differentially expressed genes
FPKM: Fragments per kilobase of transcript per million fragments mapped
HPGs: High-pressure-induced DEGs
LRGs: Linearly related DEGs
P0.1: Experimental group incubated at atmospheric pressure
P15: Experimental group incubated at 15 MPa
P25: Experimental group incubated at 25 MPa
PSGs: Pressure-sensitive DEGs
qPCR: Quantitative real-time reverse transcription-PCR.
RFC: Relative fold change
