# Supplementary figures and images for: Insights into high-pressure acclimation: comparative transcriptome analysis of sea cucumber Apostichopus japonicus at different hydrostatic pressure exposures
Source: BMC Genomics. 2020 Jan 21;21:68. doi: 10.1186/s12864-020-6480-9 (PMC6974979; doi:10.1186/s12864-020-6480-9)

**Figure S1.** Results of KEGG enrichment.

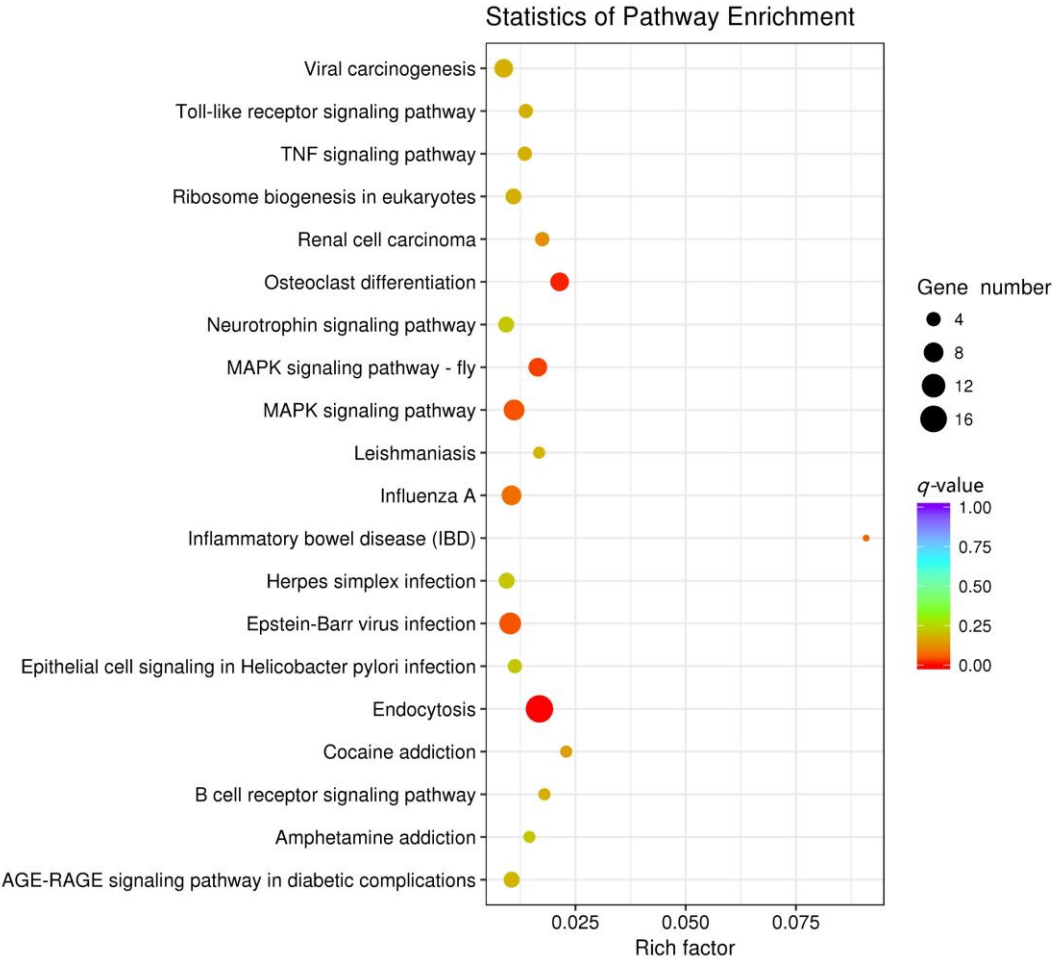

Supplement: Supplementary file 1 — Additional file 1 Figure S1. Results of KEGG enrichment. [file 12864_2020_6480_MOESM1_ESM.pdf]
