# Supplementary material for: Insights into high-pressure acclimation: comparative transcriptome analysis of sea cucumber Apostichopus japonicus at different hydrostatic pressure exposures
Source: BMC Genomics. 2020 Jan 21;21:68. doi: 10.1186/s12864-020-6480-9 (PMC6974979; doi:10.1186/s12864-020-6480-9)
Supplement: Supplementary file 2 — Additional file 2 Table S1. Quality of sequencing. P0.1: experimental group incubated at atmospheric pressure; P15: experimental group incubated at 15 MPa; P25: experimental group incubated at 25 MPa. [file 12864_2020_6480_MOESM2_ESM.docx]

**Table S1.** Quality of RNA sequencing. P0.1: experimental group incubated at atmospheric pressure; P15: experimental group incubated at 15 MPa; P25: experimental group incubated at 25 MPa.

| Sample | Raw Reads | Clean reads | Clean bases | Error(%) | Q20(%) | Q30(%) | GC(%) |
| --- | --- | --- | --- | --- | --- | --- | --- |
| P0.1_1 | 76,725,374 | 75,238,044 | 11.29G | 0.03 | 95.77 | 89.66 | 41.79 |
| P0.1_2 | 71,297,964 | 69,635,202 | 10.45G | 0.03 | 96.87 | 91.95 | 40.76 |
| P0.1_3 | 83,571,800 | 81,695,626 | 12.25G | 0.03 | 96.84 | 91.90 | 41.43 |
| P15_1 | 96,875,848 | 93,909,310 | 14.09G | 0.03 | 96.55 | 91.32 | 39.51 |
| P15_2 | 67,006,938 | 65,802,558 | 9.87G | 0.03 | 96.56 | 91.31 | 39.64 |
| P15_3 | 88,612,274 | 86,427,868 | 12.96G | 0.03 | 96.62 | 91.45 | 39.78 |
| P25_1 | 114,691,988 | 112,273,438 | 16.84G | 0.03 | 96.76 | 91.74 | 39.65 |
| P25_2 | 73,376,304 | 71,858,404 | 10.78G | 0.03 | 96.76 | 91.69 | 40.22 |
| P25_3 | 93,598,656 | 92,010,102 | 13.8G | 0.03 | 96.80 | 91.87 | 41.33 |
