# Supplementary material for: Insights into high-pressure acclimation: comparative transcriptome analysis of sea cucumber Apostichopus japonicus at different hydrostatic pressure exposures
Source: BMC Genomics. 2020 Jan 21;21:68. doi: 10.1186/s12864-020-6480-9 (PMC6974979; doi:10.1186/s12864-020-6480-9)
Supplement: Supplementary file 10 — Additional file 10: Table S9. Information of the primers used in quantitative real-time reverse transcription-PCR (qPCR) analysis and the PCC between RNA-seq and qPCR results (with cytb and ß-actin as internal control). PCC: Pearson correlation coefficients. [file 12864_2020_6480_MOESM10_ESM.doc]

**Table S9. Information of the primers used in quantitative real-time reverse transcription-PCR (qPCR) analysis and the PCC between RNA-seq and qPCR results (with *cytb* and *ß-actin* as internal control). PCC: Pearson correlation coefficients.**

| Gene name | Gene ID | PCC | 5’- 3’ forward primer | 5’- 3’ reverse primer |
| --- | --- | --- | --- | --- |
| *cytb* | Cluster-82576.285982 |  | TTCCCTAGCTTTCTCGTCAGTA | ACATTTGACCTCAAGGCAATAC |
| *ß-actin* | Cluster-82576.286401 |  | ATGAAGGATGGTTGGAAGAGGGTC | TCAAGGAGAAACTGTGTTACGTCGC |
| *AP* | Cluster-82576.303624 | 0.95 | AATGTGGTTGCCAAGTTATGCT | TCGGCATTCAACATTCTTCTTCC |
| MVP | Cluster-82576.263884 | 0.81 | GACTCCAGCGATGATGAATAGG | CAAGGACACCAAAACAAAAGAGAAT |
| CCAAT | Cluster-82576.284539 | 0.98 | ATGAACACTGATGACCAATCCCTC | TATTGCGGTTAGTTGGGACGAT |
| E3RN | Cluster-82576.260312 | 0.99 | GGGGTTGTTATTCAGGGATTGC | CCAAACGAGAGATGATAGAACGGAT |
| *IST1* | Cluster-82576.256165 | 0.97 | AATCATCCCAAACCTTGCCAGTAAC | TCTGGAGGGTATCGTTCCCAAAAGT |
| *S1* | Cluster-82576.257995 | 0.98 | TTCACAGACGAAGGTGGTTGGC | TTAGTTCTGAGGGAGGTTAGGACGC |
| *GR53* | Cluster-82576.289953 | 0.98 | TGAGGAAACCTTGCCGATTGTA | CACCCTGTAAGTCGTGAAGGATGAAC |
| *ZF36* | Cluster-82576.281154 | 0.99 | GGGGTCCGATTCCGTATTCAGT | CGATAGTGTGATATGTACGGCAGAG |
| *MRP* | Cluster-82576.301995 | 0.95 | CGGGTAGGAGACAAGGGAACAC | GGCTTGGACAACCCTTTCACTT |
| HT | Cluster-82576.229549 | 0.97 | CCTAAGAAGTCATTATGTCGCTGGT | AAAGCTAGTACCTGGTCATCCTCGT |
| N1 | Cluster-82576.269386 | 0.97 | CAGATGTAGATGCTGCCGAGGTATT | CGAGCAGCGGTGATTCCAAGTA |
| *NRC* | Cluster-82576.223787 | 0.93 | CAGTGCTGATGTAGAGTGGACGGTA | AGCCACTGCTTATGACCGACCT |
| CMB | Cluster-82576.288817 | 0.99 | ACCTCGTCCGTCATTTCCAACA | GGAGCAAGCCAACTACGCCACA |
| *UC* | Cluster-82576.334133 | 0.99 | TGCCAATGTGACAAAGAACCGAGAT | CGAACACGGAAGACTCAATCAACAG |
